# Evidence of co-creation practices in suicide prevention in government policy: a directed and summative content analysis

**DOI:** 10.1186/s12889-022-14313-3

**Published:** 2022-10-17

**Authors:** Tania Pearce, Myfanwy Maple, Sarah Wayland, Kathy McKay, Anthony Shakeshaft, Alan Woodward

**Affiliations:** 1 School of Health, University of New England, 2351 Armidale, NSW Australia; 2 Public Health, Policy and Systems, Institute of Population Health, University of Liverpool, Liverpool, UK; 3 Tavistock and Portman NHS Foundation Trust, London, UK; 4 National Drug and Alcohol Research Centre, University of New South Wales, Randwick Campus, 22-32 King Street, 2031 Randwick, NSW Australia; 5 Centre for Mental Health, School of Population and Global Health, University of Melbourne, VIC, 3010 Melbourne, Australia

**Keywords:** Co-creation, Government policy, Mental health, Suicide prevention, Co-design, Qualitative content analysis

## Abstract

**Background:**

In Australia, the collaborative involvement of stakeholders, especially those with lived experience in mental health and suicide prevention, has become important to government policy and practice at Federal and State levels. However, little is known about how governments translate this intention into frameworks of co-creation for policy, funding programs, service improvement, and research and evaluation. We investigated the extent to which publicly available government policies refer to collaborative practice using an established translation model.

**Methods:**

An exploratory directed and summative content analysis approach was used to analyse the contents of Federal (also known as Commonwealth), State and Territories policy documents on mental health and suicide prevention published in Australia between 2010 and 2021. The data was extracted, compared to an existing translation model, and summated to demonstrate the evidence of co-creation-related concepts between government and stakeholders.

**Results:**

40 policy documents (nine at the Federal and 31 at the State and Territory level) were identified and included in the analysis. Only 63% of policy documents contained references to the concept of co-design. Six of the State policies contained references to the concept of co-production. Across all policy documents, there were no references to other concepts in the model adopted for this study, such as co-creation, co-ideation, co-implementation, and co-evaluation.

**Conclusion:**

Although the government at Federal, State and Territory levels appear to support collaborative practice through partnership and co-design, this study suggests a narrow approach to the theoretical model for co-creation at a policy level. Implications for both research and practice are discussed.

## Introduction

In Australia, mental ill health and suicide cost the community between $43 billion and $70 billion annually [1, 2], while the estimated national expenditure on mental health services in 2018–2019 amounted to $10.6 billion [3]. Meanwhile, annual deaths from suicide in Australia stand at 12.1 per 100,000 people [4], with rates of attempted suicide and suicidal ideation on the rise [5]. Suicide and suicidal behaviour remain significant economic and epidemiological burdens in Australia, leading researchers, organizations, and government agencies to seek innovative approaches and practical solutions when addressing these ongoing mental health and suicide issues. One creative strategy involves governments and researchers engaging with consumers and carers to develop mental health policy and improve how services are delivered. The promotion of user involvement or a person-centered approach comes from the “nothing about us without us” [6] and the “sit beside me, not above me” [7], both of which promote greater carer and consumer involvement in the decision-making process. Alongside the increased participation of users in the planning and delivery of mental health and suicide prevention services, multisectoral collaborations between government, researchers, service providers, and users are also becoming increasingly significant. It is argued that multisectoral collaborations may resolve complex issues such as suicide prevention more effectively than researchers alone [8]. For instance, translation frameworks such as co-creation of new knowledge are a current example of how stakeholders (researchers and other stakeholders, including those with lived experience) may collaboratively engage in program evaluation through four collaborative processes, that is, *i) generating an idea (co-ideation); ii) designing the program or policy and the research methods (co-design); iii) implementing the program or policy according to the agreed research methods (co-implementation), and iv) the collection, analysis and interpretation of data (co-evaluation)*” [9]. For governments, several benefits come from increased stakeholder participation in research and service delivery. For instance, engaging stakeholders in the design phase may result in mental health and suicide prevention services meeting the needs of stakeholders [10].

Additionally, involving stakeholders in the research process will increase stakeholders’ participation, particularly if the research impacts policies that directly affect them [11]. Collaborations between researchers and consumers may also improve service quality and outcome effectiveness by evaluating suicide prevention programs. In turn, this can enhance the development of sustainable research and innovation [12, 13]. Despite such promises, it is unclear whether mental health and suicide prevention policies reflect ideas on person-centered participation and multisectoral collaboration. Remarkably, there is little clarity about whether collaboration between stakeholders impacts research outcomes and funding and how it is measured. Optimising the impact of collaboration is critical, given that policy and funding remain primary drivers in the development of mental health and suicide prevention strategy and the services delivered through community organisations [14]. Identifying any gaps in policies that may limit the implementation of effective collaborative practices will improve understanding of how the existing policies and their scope for action are likely to be used.

A complete examination of Federal, State and Territory policy documents is needed to understand how collaborative processes involving stakeholders, especially those with lived experience, are represented through mental health and suicide prevention policies. The study will reference an existing peer-reviewed theoretical framework, “co-creation of new knowledge,“ to compare critical elements associated with collaborative processes within the research cycle [9]. Our knowledge indicates that no published research has previously examined mental health and suicide prevention policies in co-creation or collaborative processes.

The following aims were formulated in conjunction with the authors, who identify themselves as either a researcher, service provider, a person with lived experience, or a combination of roles. Specifically, the study has four aims: (1) identify mental health and suicide prevention policies published between 2010 and 2021; (2) capture the frequency of keywords and compare them across identified policy documents (3) describe links between mental health and suicide prevention funding and the principles of co-creation and; (4) assess how policies prioritise four co-creation related activities (co-creation of new knowledge), and other collaborative activities. This study addresses the critical debate on the gap.

## Materials and methods

### Content analysis

The research questions were addressed using content analysis methodology. By definition, content analysis is a qualitative descriptive methodology used to make “valid inferences from verbal, visual or written data in order to describe and quantify specific phenomenon“ [15] (p.18). It is beneficial for studies where the purpose is not to collect rich descriptions of the findings but to detect patterns or outliers within qualitative data [16].

### Directed and summative content analysis

For this study, we chose a combination of directed and summative content analysis, two well-known approaches used in content analysis. This approach has been commonly used for the research of documents requiring a low level of interpretation [17], such as health guidelines [18] and policy documents [19]. Specifically, a directed content analysis involves using a set of pre-defined codes (deductive coding) created from an existing theory or framework to categorise data [20]. The application of deductive coding increases the likelihood that both manifest content (observable and direct representation of specific words) and latent content (underlying representation and interpretation of concepts) are captured [21]. Meanwhile, for summative content analysis, the frequency of specific words appearing in the text are counted and compared across coding categories, followed by further analysis to interpret the context of the frequency of words [20]. A directed content analysis was used for Aims 2 and 3, while a summative content analysis was used to address Aim 4.

### Eligibility

We defined health policies as documents meeting the following three principles: (1) authored by a governing body ( elected to exercise authority), (2) a document that outlines the objectives, strategies, or goals, and, (3) includes the planning, organisation, delivery or improvement of services, programs or strategies [28]. In Australia, policy documents are labelled using a variety of terms, including “strategy”, “policy”, “strategic plan”, “plan”, “strategic framework”, “action plan”, “framework” and, “report”. To identify relevant mental health and suicide prevention policy, we used the following inclusion criteria: (1) satisfy the definition of a policy document where an Australian governing body authors documents at the Federal, State or Territory level, (2) policy documents had to have a primary focus on issues relating to mental health and/or suicide prevention, and (3) policy documents were limited to those published after January 2010. The period of January 2010 to October 2022 was determined following a pilot search of the literature where co-related type terms (e.g., co-design, co-production) began appearing in the academic literature alongside suicide prevention and mental health.

### Searching and screening

From 1 to 2021 to 15 October 2021, we completed three discrete rounds of searching to identify publicly available and relevant policy documents on mental health and suicide prevention. The first round targeted Australian government health websites at the Federal, State and Territory levels. The second round involved a title and abstract search of Trove, the National Library of Australia (NLA’s) [22] online library database aggregator, along with a grey literature search using Google and Google Scholar. These two searches were optimised by a third manual search of policy documents. This third process involved scanning policy documents retrieved in the first and second rounds of searching for references to additional policies. Keywords used to search the databases and websites included “mental health” and “suicide” with searches limited to those publications published after January 2010 and websites using Australian government domain names “.gov.au”.

Our quality assurance process included checking all identified policies against Mindbank, a database maintained by the World Health Organization [23], which lists health policies by country and specialty, including suicide prevention, and asking three experts to review the final list and identify any missing policies.

### Data extraction

All identified documents retrieved from online searches were imported into Endnote X9, where a reference library had been created to allow storage and management of full-text documents. Following this step, the full-text versions of identified documents were then exported to NVivo 12 Pro QSR, a qualitative analysis software.

### Data analysis

#### Deductive analysis

For the deductive analysis, we chose to analyse manifest and latent representations of terms relating to the co-creation of new knowledge framework. As briefly described in the introduction, co-creation of new knowledge is a translation model which works alongside the delivery of health interventions such as suicide prevention programs [9] and relies on the collaboration between researchers, third sector organisations, and those with lived experience to generate new knowledge. Through this process, stakeholders engage in five collaborative processes these being co-creation, co-ideation, co-design, co-implementation, and co-evaluation. Since the aim was to identify the usage of these “co” processes within policy documents, the co-creation of new knowledge framework, as presented in Table 1, guided the makeup of categories used in NVivo 12 Pro QSR.

**Table 1:** *Co-creation coding framework*

	Core Principles	Definition
Co-creation	Co-Ideation	Engaging in open dialogue to share new and creative ideas for the solving of problems relating to new products, services, policies, and programs
	Co-Design	Describing the technical details of new products, services, procedures, policies, or programs (prototype), as well as the research methods to be used (protocols). This process may include assessment of funding sources, availability of resources, research processes (e.g., ethics), and timelines.
	Co-Implementation	Implementing the co-designed program, policy or clinical procedures by following the research protocol. This process may be a one-time collaborative event or an arrangement over the longer term.
	Co-Evaluation	Embedding data collection or other formal research techniques into the co-implementation process. Researchers with relevant bio-statistical skills undertake analyses. Co-interpretation of the meaning and implications of the results.

To expedite the data analysis process, we used the text search function in NVivo 12 Pro QSR to search policy documents for the five co-related processes.

#### Inductive analysis

We used a manual open coding process for the inductive analysis to identify the five co-creation-related domains. This process involved the lead author becoming familiar with the data through careful reading and re-reading of the documents and manually coding text relating to the co-creation process by highlighting manifest or latent phrases or segments of data. During the manual coding process, the data was categorised into themes and subthemes, which, over time, were reviewed and refined to represent ideas and patterns of meaning. Emerging themes and sub-themes were discussed with authors KM and SW and were further refined through this discussion. Data on the coverage or frequency of terms was collated using NVivo 12 Pro QSR and converted into a heat map using Microsoft Excel. The coverage of terms indicates how often categories of co-creation and related concepts were cited across mental health and suicide prevention policy documents. Coverage data provides insight into the significance of specific terms. Therefore, the higher the level of coverage of a concept or term, the higher the rate of interest in or discussion of that term across the documents. For terms appearing in multiple policy documents published in the same year, the average of the coverage rate was reported. In addition to the coding process and the coverage data, a data extraction form was developed in Excel to capture critical information on policy characteristics, including the name of the policy, year, level of government (Federal, State or Territory), and policy focus (mental health or suicide prevention).

Trustworthiness.

The trustworthiness of the content analysis was evaluated using Lincoln and Guba’s [24] four standards (credibility, dependability, transferability, and confirmability) evaluation criteria. Credibility was achieved by including sufficient detail about the data analysis process and using a process of systematically comparing categories to ensure consistency of the data had been maintained. Dependability was demonstrated by maintaining clear documentation about the process used to collect data, and the development of the coding frame was reviewed by three of the co-researchers. Meanwhile, transferability was reached by ensuring all relevant Federal, State or Territory policy documents on mental health and suicide prevention were included. At the same time, the data was confirmed through feedback from several co-authors, all of whom are experts in mental health and suicide prevention. In addition, confirmability was further attained through an audit trail whereby tables and results demonstrate transparency of the data collection and analysis.

## Results

### Identification of mental health and suicide prevention policy documents

We searched the literature and identified 40 unique mental health and suicide policy documents meeting the study inclusion criteria. Nine related to Federal policies [2, 8, 25–31] while the remaining 31 documents represented the following Australian States and Territories: New South Wales (NSW) (n = 8) [32–39]; Northern Territory (NT) (n = 4) [40–43]; Queensland (QLD) (n = 4) [44–47]; Western Australia (WA) (n = 4) [48–51]; South Australia (SA) (n = 4) [52–55]; Tasmania (TAS) (n = 4) [56–59]; Victoria (VIC) (n = 2) [60, 61], and Australian Capital Territory (ACT) (n = 1) [62]. The field of mental health was the focus of one Federal policy [27] and 12 State policy documents [32, 37, 39–41, 44, 46, 48, 52, 53, 58, 60], while four Federal [8, 25, 26, 31] and 17 State and Territory policy documents [33–36, 38, 42, 43, 45, 47, 49–51, 54–57, 61] were solely dedicated to suicide prevention. The remaining six policy documents (Federal n = 5; Territory n = 1) covered mental health and suicide prevention [2, 27–30, 62].

### Identification of keywords in text analysis

Table 2 provides the results of the deductive and inductive analysis, including the number of references for each term and exemplar quotes to demonstrate the results presented. Of the group of terms relating to the co-creation framework listed in Tables 1, only “co-design” was cited. Meanwhile, domains identified through inductive coding generated an additional six categories of terms frequently used in conjunction with co-creation of new knowledge. These included “collaboration”, “funding”, “research and evaluation”, “stakeholders (including lived experience)”, “Third Sector Organisations” and “co-production”. The most frequently cited terms were “collaboration” (n = 637) and “funding’ (n = 628). The next most frequently cited terms were “stakeholders” (including Lived Experience)” (n = 408), “research and evaluation” (n = 350) and, “Third Sector Organisations” (n = 236). Co-production was cited the least across all policy documents.

**Table 2:** *Summary of co-creation related domains and frequency of references*

Co-creation Domains Frequency of References (n=)	Extraction Criteria	NVivo Search Terms using Boolean “OR” operator (Truncation used where applicable and with and without hyphens)	Exemplar
Collaboration (n = 637)	Any manifest or latent mention of collaborative partnerships between researchers, service providers and/or service users	Collaboration, collaborative, etc. partnership, “work with”	*“The system will be co-designed with a collaborative approach across communities incorporating both lived and professional experience.” (*30*)*
Funding (n = 628)	Any manifest or latent references to Government funding directed towards programs, service providers and service users or acknowledgment of the importance of long-term funding, or evidence of the link between funding and research and evaluation	fund	*“Enable long term funding cycles to facilitate consistency, sustainability and quality improvement. “(*30*)*
Stakeholders, including Lived Experience (n = 408)	Any manifest or latent references to any group or individual who is affected by or can affect the achievement of an organisation’s objectives (Freeman,2001), including policymakers, service providers and/or service users/consumers	Stakeholder, “lived experience”, “peer worker”, “peer workforce”, consumers, carers	*“include the wisdom of those with a lived experience into research, policy and service development.” (*50*).”*
Research and Evaluation (n = 350)	Any manifest or latent references to the planning of research and evaluation of services and programs or acknowledgement of the importance of research and evaluation in improving health and societal outcomes	Research, evaluation	*“evaluation is critical for creating a stronger evidence base to drive continuous improvement in suicide prevention policy, services and programs.” (*53*)*
Third Sector Organisations (TSOs) (n = 236)	Any manifest references to Third Sector Organisations, NGO’s or Non-Profit Organisations (NPO) and the role they play in service provision, partnerships with other stakeholders, or participation in research activities	“TSO”, “Third Sector”, “NGO”, “non-government”, “non-profit,” “Not for Profit”	*“building stronger partnerships between government and non-government organisations is critical to supporting those at risk of and impacted by suicide.” (34)*
Co-Design (n = 107)	Any manifest or latent references to co-design where stakeholders participate in the design of a new program or product	Co-design	*“innovative co-design approach, more than 2100 people came together either online or in-person to develop ideas and comment on working papers” (39)*
Co-Production (n = 22)	Any manifest references to stakeholders co-producing the design, development, and delivery of services	Co-production	*“government will co-produce policy and services with people with mental illness, their families and carers, and clinicians and other mental health workers” (39)*
Co-Creation, Co-Ideation, Co-Implementation, Co-Evaluation	Any manifest or latent references to these terms indicating participation by stakeholders in the research process, whether in part or as a whole (co-creation)	Co-creation, co-ideation, co-evaluation, co-implementation	*No Examples Available*

#### Co-design and co-production

Across 25 Australian policies, six Federal [2, 25, 27, 29–31] and 19 State and Territory documents [32, 33, 35–39, 41, 45–47, 49–51, 53, 57, 60–62], there were 107 references to the word “co-design” (and its variants including co-designed and co-designing). Overall, nine policies [2, 25, 27, 30, 32, 35–37, 60] offered definitional descriptions of co-design, with one NSW policy [32] perceiving the concept as a tool for services where co-design is used: *”to work collaboratively with staff, consumers, families and carers on redesigning mental health services to prevent suicides among people under care”* (p.17), and as an approach to assist “*services to deliver person-centred care through considering consumer, carer, staff and other stakeholder perspectives in planning and service delivery*” (p.129). The same policy also chose to describe co-design in terms of the individuals involved and benefits, for example:

“*Co-design…..brings together the expertise of people with a lived experience of a suicide attempt or who have been bereaved by suicide, families and carers, service providers, key stakeholders and community groups to produce an outcome which is mutually valued across the community*” [32] (p.21).

Meanwhile, the NSW Aboriginal and Mental Health and Wellbeing Strategy 2020–2025 [37] defined co-design in the context of health services as “*a collaborative approach…to improve health services*. *In co-design, the people who use and deliver health services are deliberately engaged to share experiences and collectively imagine and create solutions that innovate, change and improve health services*” (p.13). In analysing co-design, the authors observed no discernible trend in discussions about (i) co-design in connection with policy aims; (ii) how organisations, such as TSOs, might engage in co-design; or (iii) guidance on the potential benefits or challenges of such a collaborative process. According to the documents, co-design is a best practice model for developing tailored mental health and suicide prevention services that meet the needs of individuals and their families. Meanwhile, co-production appeared in six policy documents [32, 49–51, 53, 60], wherein co-production was used as a synonym for co-design. For example, an extract from Victoria’s 10-year mental health plan [60] describes co-production as a collaborative process where:

“*government will co-produce policy and services with people with mental illness, their families and carers, and clinicians and other mental health workers. People will have a genuine say about how the system works, how services work and how they are treated. The result will be services that work much better for the people they serve*” (p6).

#### Collaboration

The concept of collaboration was the most commonly used co-creation-related term across all 40 policy documents, with 637 manifest or latent references identified. In 12 documents [29, 31, 34–36, 39, 41, 42, 52, 56, 60, 62], references to collaboration were made in the broader context of a “*whole of government”* or a “*whole of community*” approach. These approaches characterise the forming of strong, co-ordinated partnerships between all sectors of government, and stakeholders, including researchers, TSOs, carers and consumers, to strengthen communities and improve suicide and mental health initiatives. For instance, at a Federal level whole of government approach is seen to “*unlock the potential of a whole of government delivery model by ensuring each individual agency has strong processes and accountabilities for delivering agreed suicide prevention initiatives, and linking into broader collaborative efforts across government*” [31] (p24). Meanwhile, policies view “*a whole of government, whole of community approach*” [35] as a formal linking of activities “*that places greater emphasis on integration and collaboration between all levels of government, individuals and communities, the non-government and private sector, and people with lived experience*” [35] (p2). Across policies, collaboration was generally described in favourable terms espousing the benefits partnerships provide towards improving suicide and mental health outcomes. For instance, *“The role of carers and consumers in supporting and informing intersectoral collaboration will be essential at all levels of policy, planning, research, service development and delivery in order to ensure the best possible health outcomes”* [52] (p14) and, *“growing body of evidence shows that services designed in collaboration with those who use them are more efficient and less expensive”* [39] (p47). A strong emphasis was also placed on collaboration and equity by one state policy wherein: “*Research shows that giving people an equal voice as active partners in healthcare improvement can lead to better experiences and outcomes for all. A key to improving outcomes is respecting the expertise of consumers, carers and staff in guiding individual recovery as well as co-design*” [32] (p82). Latent examples relating to the idea of collaboration between consumers and carers used terms such as consultation and engagement to describe the collaborative process between consumers and carers “*Supporting consumers and carers to effectively engage and participate will remain a key focus of the NMHC’s work. This will include consultation and engagement on a range of issues, from an individual accessing mental health services, to the contribution of consumers and carers to mental health service planning, delivery and engagement on mental health reforms”* [2] (p9).

#### Stakeholders (including lived experience)

The term “stakeholders” includes references to “lived experience” (also known as consumers or peer workers), featured in 34 policy documents with 408 manifest or latent mentions. In all of the documents, the concept of stakeholders extended to include individual groups such as “*those impacted by suicide, researchers, non-government service providers and State Government agencies*” [47] (p3), with their role defined as working “*collaboratively to ensure a comprehensive and coherent approach to legislation, policy, planning, funding and service delivery”* [46] (p16). References to stakeholders were significantly focused on establishing “*equal partnerships*” [46] between stakeholders and mental health consumers. In this context, those with “lived experience” were seen to have “*a valuable, unique and legitimate role in suicide prevention*” [49] (p10) and an essential factor in creating change through research and practice “*we must position lived experience knowledge at the forefront of research, policy and practice. Without it, our reforms and service improvements will fall short of what people need and what they deserve*” [8] (p2). In 13 policies [25, 27, 32, 33, 35, 37–39, 45, 50, 51, 53, 60], the role of mental health consumers shared a strong connection with co-creation related activities such as “co-designing” programs and services, as evidenced by statements such as “*the development and implementation of suicide prevention strategies must include their voices, and activities should be co-designed with people with a lived experience*” [50] (p2). There were eight policies [8, 25, 26, 31, 39, 46, 50, 51] referencing the integral participation by Indigenous or cultural groups as stakeholders in the planning of programs and services “*Governance must also incorporate early input from the portfolio’s priority populations to ensure approaches are relevant, respectful and effective. This includes cultural governance inclusive of Indigenous people and integrating people with lived experience into planning and advisory stages*” [31] (p27) and “*The insights of people with lived experience of suicide; traditional forms of knowledge, such as from Aboriginal people and unique cultural perspectives, can form part of the evidence base for effective suicide prevention. Continual development, implementation and evaluation of existing and future initiatives is crucial”* [50] (p11). Meanwhile, other examples highlighted the importance of Indigenous involvement in co-design and service delivery but failed to explain how such an approach might work. For instance,

“*Aboriginal people are experts in Aboriginal communities and needs, and that improvements in the coordination of services and in the quality of service delivery and planning will need to start in genuine co-design processes, led by Aboriginal people. Person centred and culturally safe services acknowledge the strength and resilience of Aboriginal people, families, and communities”* [37] (p10).

#### Third sector organisations

The concept of “Third Sector Organisations” and related terms such as “non-government” and “non-profit” appeared in 236 references across 34 policy documents. In one suicide prevention strategy document, TSOs were identified as a type of stakeholder who worked collaboratively with other actors: “*Suicide prevention is complex – and it is everyone’s business. A coordinated, well-integrated and compassionate approach is required across all levels of government and from the community, including individuals, families, schools, researchers, community groups, non-government services and the private sector*” [35] (p7). While there was evidence of government support for the contribution TSOs make, for instance, “*Government also recognises the significant achievements of the non government sector in suicide prevention to date, and acknowledges that building stronger partnerships between government and non government organisations is critical to supporting those at risk of and impacted by suicide*” [34] (p1). There was also explicit pressure on TSOs to demonstrate effectiveness and performance measures, where it was suggested, “*Tie receipt of ongoing Australian Government funding for government, NGO and privately provided services to demonstrated performance*” [2] (p53). Only three policies mentioned TSOs and participation in research [26, 34, 51] with strategies proposing the development of “*options for prevention research partnerships between the community sector, non-government organisations and research and training sectors to build capacity in suicide prevention”* [26] (p38). Surprisingly, besides a brief mention of TSO participation in a co-design workshop [35], policies contained no explicit or implicit references connecting TSOs and engagement in co-creation-related-activities such as “co-design”.

#### Research and evaluation

The terms “research” or “evaluation” appeared in 39 of the 40 policy documents, generating 350 references. Notably, manifest or latent references to co-creation activities such as co-design or co-production were infrequently discussed in close proximity to concepts of research and/or evaluation (n = 14) [25, 27, 30–32, 37, 40, 41, 45, 47, 52, 54, 56, 61]. In these cases, co-design (or co-production) was only described in general terms and there were no explicit or implicit references on how co-design could be incorporated into research and evaluation. For instance, when referring to reforming the mental health system, one policy implicitly stated:

“*Collaborative partnerships with consumers and carers are integral to successfully implementing changes that improve outcomes for people with, or at risk of, mental illness and/or suicide. Examples of supporting ongoing and active involvement of consumers and carers include collaboration on design and planning, implementation, monitoring and evaluation”* [27] (p49),

while a Federal policy referencing a national person-led system asserted, “*The system will include capacity building and tools for modelling, need analysis, co-design, implementation and evaluation”* [30] (p21).

#### Mental health and suicide prevention funding and links to principles of co-creation

A search of all included policy documents revealed no discernible evidence of government declarations of support for research and evaluation of co-creation-related activities. However, among the manifest references where co-design was associated with funding, we identified two references: “*Funders need to ensure they are supporting the ACT mental health workforce, including they are engaged in co-design of system reform*” [62] (p32), and *“$1.1 million to the Black Dog Institute to work with the Aboriginal and Torres Strait Islander Lived Experience Centre, supporting the inclusion of people with lived experience in the co-design, implementation and evaluation of suicide prevention activity*” [25](p19). Overall, funding-related references were associated with the funding of services [62], how funding was sourced [27], and funding models [53]. In other examples of discussions around funding, there was criticism of how the failure of evaluation and funding leads to poorly planned assessments of interventions. For instance, in a strategic plan published by the Mental Health Commission of NSW [39], it was stated: “*While all funded initiatives are required to have an evaluation component, evaluation requirements are not always rigorous enough and funding is not always sufficient for meaningful evaluation, which limits their contribution to the evidence base*” (p 37). References emphasising the importance of research and evaluation could be found in a list of Federal standards and quality in suicide prevention for Aboriginal and Torres Strait Islander communities [26], where it was noted that,

“*Provision for evaluation can be significantly improved in funding arrangements under state and Commonwealth contracts. There are currently very few evaluations conducted that contribute to the evidence base in any way. Aboriginal and Torres Strait Islander community services benefit from evaluations of programs that demonstrate their effectiveness and that provide information for practice development, policy and planning*” (p44).

In the same document, emphasis is put on ensuring that “*suicide prevention principles are embedded in systems of quality improvement for social and emotional wellbeing and mental health care*” [26] (p44) while failing to include the embedding of rigorous research methods or data collection into service delivery. Meanwhile, of all State policies, the South Australian Suicide Prevention Strategy 2012–2016 [54] was the most explicit in its approach stressing the importance of linking funding with evaluation: “*State funded programs to be evaluated prior to funding renewal*” and “*All suicide prevention programs be properly evaluated with at least 15% of all funding allocated to suicide prevention programs being spent on evaluation*.” (p44).

#### Coverage of terms across policy documents

The density of coverage (darker shade represents greater frequency) of terms across Federal, State, and Territories (Table 3) by publication year are depicted as heat maps. Coverage of co-design, for instance, was strongest in 2020, while the use of terms relating to “collaboration” peaked in 2018. In 2012, across both Federal, State and Territory policies, the term “research” attracted the most coverage, while discussions relating to stakeholders were most prominent in 2020. While references to TSOs were highest in 2010, there was a drastic decline in discussions of TSO in suicide prevention, suggesting interest by the government in TSO-led suicide prevention services had waned over the following decade.

**Table 3:**
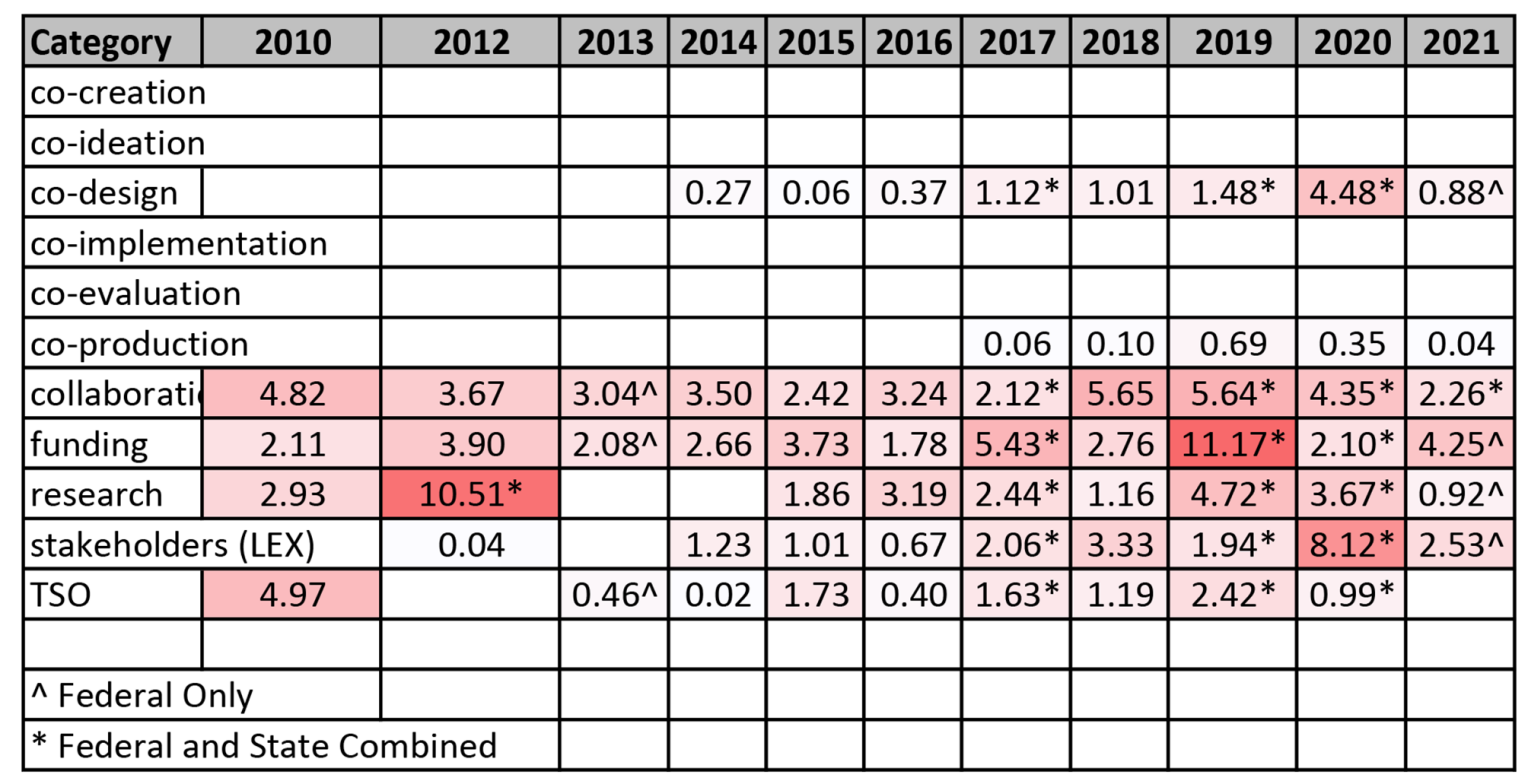
*Heat map representing code coverage statistics of terms across Federal, State, and Territory suicide prevention policies*

## Discussion

This study identifies a gap between publicly espoused policy directions and actual practice. Specifically, we found that the main focus for suicide prevention policy was on co-design and, to a lesser extent, co-production as a form of collaborative practice between stakeholders. The government views these two constructs as the driving factor in the collaborative planning, design, implementation, and evaluation of mental health and suicide prevention projects. The government considers co-design a tool for bringing relevant groups of people together to make the design of programs and services more efficient and effective.

However, no terms relating to co-creation were identified apart from co-design, with co-production being a term that sits outside of the co-creation model. This is an important detail as co-creation of new knowledge represents a translation model which works to ensure investment by stakeholders in the research process. Furthermore, there was no evidence of discussion around the use of robust or rigorous research methods in these collaborative activities. Perhaps this represents an assumption on behalf of the government that rigorous evaluation will be incorporated into practice without it being explicitly stated in policy. However, as evidenced by a report on the evaluation of Indigenous programs, only 6% (3/49) of programs utilised rigorous methods, and of those, none met the criteria of gold standard (Randomised Control Trials (RCTs) [63].

Second, our analysis of references to “third sector organisations or non-government organisations” shows the intent to describe the role of TSOs in mental health and suicide prevention using broad, sweeping statements. Across the 40 documents, there is no substantial evidence of a link between TSOs and the concepts of co-creation. It is clear that TSOs are essential in delivering support services and collaborating with a wide range of stakeholders, including those with lived experience, primary health networks, and government agencies. However, no description of how this collaboration will be managed or how it looks from a practical standpoint is provided. For policymakers, TSOs’ roles were defined in terms of service delivery rather than equitable participation in research. Consumers, carers, and people with lived experience, however, were seen as integral to research and evaluation. With an inherent lack of activity and broad references to collaboration, the inclusion of TSOs could be interpreted as tokenistic.

We uncovered three critical disconnects. First, besides offering definitions and characteristics of co-design, policies offered little guidance on how communities, like TSOs and those with lived experience, might implement co-design into suicide prevention initiatives. Policies presented no monetary encouragement for communities and organisations to engage with collaborative processes like co-design (even though throughout all of the policies, collaboration between carers, consumers or lived experience, TSOs, and other stakeholders, were strongly promoted). Second, there were no explicit or implicit references regarding the role of researchers when collaborating with those with lived experience or TSOs, even though keywords such as research and evaluation were frequently mentioned throughout the included policy documents. Third, the policy research gap remains an ongoing challenge. Although this paper’s findings indicate support for collaborative practice and co-design, a recent systematic review observed no discernible trends relating to multisectoral collaborations or co-creation-related activities, including co-design in suicide prevention interventions [64]. These disconnects in policy implementation arguably impact how effective and appropriate collaboration can be undertaken between researchers and other stakeholders. The benefits of multisectoral collaboration should be considered, given the high emotional, social, and economic costs of suicidal behaviours and the need to ensure that the prevention and intervention services provided can support the communities they claim to target.

In synthesising our findings, there are two key considerations for future policy development should collaborative practices continue to be espoused as important to service development funded through government avenues. First, linking funding to the co-creation activities, specifically by including people with lived experience, TSOs, and researchers throughout the cycle. For this to be fully embedded in policy, funding and reporting must be linked to these activities. Second, inconsistent terminology leads to confusion about the importance of different tasks. The issue of “conceptual ambiguity” around co-related terms makes it “difficult for service providers and policymakers to engage in co-creation activities because they are being asked to engage in a process that either lacks clarity or is highly variable across different researchers and disciplines” [9]. For planning, describing, and evaluating, it is therefore essential that universities and industry, e.g., researchers and TSOs, distinguish between co-creation and co-design.

### Strengths and limitations of the study

At the time of writing, the research team is unaware of other published studies examining the presence of co-creation in policies on mental health and suicide prevention. Examining how these practices are, or are not, embedded within the policy sphere is a way of understanding the importance placed on these activities by the main funding bodies of health and human services in Australia. A further strength is using both summative and directed content analysis to collect manifest and latent data as frequency counting of keywords. This approach provided a holistic approach to interpreting the issues specific to mental health and suicide prevention policy documents [65].

Among our limitations were the eligibility criteria and the definitions of policy documents. Most of the documents included in this study represent early-stage policy documents or plans. Therefore, they are not manifestations of policy action or implementation. A further limitation is our sole reliance on policy documents, whereas we could have supplemented our understanding of policy context by introducing alternative perspectives through qualitative interviews with government representatives.

## Conclusion

An examination of 40 mental health and suicide prevention government policies over a 10-year period have revealed continuous commitment by the Australian Federal, State and Territory governments to include concepts such as lived experience and co-design in suicide prevention. However, a detailed examination of these policies reveals that lived experience and co-design are oversimplified terms that fail to capture the complexity of implementing and evaluating these programs and what they mean in the context of suicide prevention. The importance of a comprehensive approach to the co-creation of new knowledge is yet to be realised. While the broad policy intent around collaboration is welcomed, if this is not reinforced through policy references to the range of processes and practices surrounding co-creation, there will not necessarily be the depth and range of stakeholder, lived experience, researcher and community involvement required for success. This narrow policy orientation around co-design and co-production may restrict the potential for policy, program, and service improvements in mental health and suicide prevention.

## Data Availability

The data used to support the findings of this study are available on request from the corresponding author.

## Abbreviations

(ACT): Australian Capital Territory.
(LEX): Lived Experience.
(NLA): National Library of Australia.
(NSW): New South Wales.
(NT): Northern Territory.
(QLD): Queensland.
(SA): South Australia.
(TAS): Tasmania.
(TSOs): Third-Sector Organisations.
(VIC): Victoria.
(WA): Western Australia.
(WHO): World Health Organization.
